# Combination of CIK cells with oncolytic viruses: A potential Immunovirotherapy strategy for the treatment of solid tumors

**DOI:** 10.1016/j.tranon.2026.102996

**Published:** 2026-08-28

**Authors:** Milad Parvinzad Leilan, Mona Arianejad, Behnam Omidi Sarajar, Gholamreza Tavoosidana

**Affiliations:** a Department of Molecular Medicine, School of Advanced Technologies in Medicine, Tehran University of Medical Sciences, Tehran, Iran; b Department of Toxicology and Pharmacology, Faculty of Pharmacy, Tehran University of Medical Sciences, Tehran, Iran

**Keywords:** CIK cells, Oncolytic viruses, Combination therapy, Solid tumors, Cancer

## Abstract

Cytokine-induced killer (CIK) cells represent a promising cellular immunotherapy, characterized by potent cytotoxicity, major histocompatibility complex (MHC)-unrestricted tumor recognition, and ease of *ex vivo* expansion. Despite these properties, their clinical efficacy against solid tumors remains limited by poor tumor infiltration, limited *in vivo* persistence, immunosuppressive mechanisms with in the tumor microenvironment (TME), and physical barriers imposed by the dense extracellular matrix (ECM). Oncolytic viruses (OVs) are being investigated as anticancer agents for their tumor-selective replication, induction of immunogenic cell death, and capacity to remodel the TME. OVs can disseminate within solid tumors and penetrate tumor tissue more effectively than cell-based therapies alone. Beyond direct oncolysis, OV-induced tumor cell death promotes systemic antitumor immunity through the release of tumor-associated antigens (TAAs) and damage-associated molecular patterns (DAMPs). Preclinical studies further suggest that CIK cells can serve as cellular vehicles for OV delivery, protecting viral particles from premature immune clearance and improving intratumoral viral replication and spread. On this basis, combining OVs with CIK cells offers a mechanistically coherent strategy that couples OV-mediated oncolysis and immune activation with the cytotoxic function of effector immune cells. This review explores the molecular rationale for CIK-OV combination immunovirotherapy, summarizes current preclinical and clinical evidence, and discusses the opportunities and limitations of this combinatorial approach for solid tumor treatment.

## Highlights

• The integration of CIK cells with OVs represents a cell-based combination strategy with the potential to generate additive or synergistic antitumor effects.
• Oncolytic viruses can remodel the tumor microenvironment and enhance immune cell infiltration.
• CIK cells may improve the targeted delivery and intratumoral persistence of oncolytic viruses.
• The combination of CIK cells with OVs shows promise for solid tumors but requires careful translational optimization.

## Graphical abstract

**Figure:**
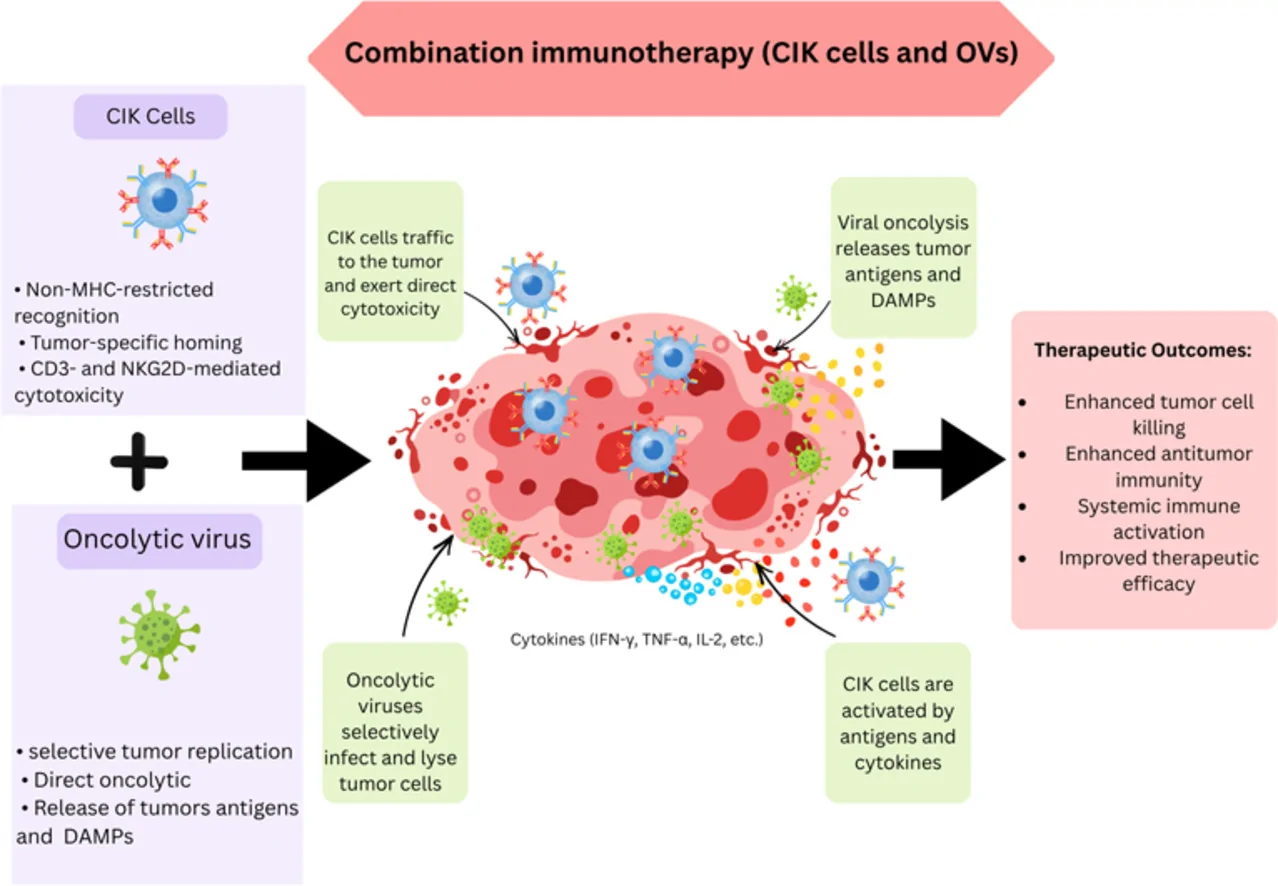

## Introduction

One of the emerging anticancer therapeutic strategies is harnessing the immune system to recognize and eliminate malignant cells. This approach has been widely recognized as an effective strategy for cancer treatment. The first documented success of cancer immunotherapy dates back to the 1890s, when William Coley employed microbial toxins derived from *Streptococcus erysipelatis* and *Bacillus prodigiosus* bacteria to treat patients with cancer [1]. Our understanding of fundamental immune processes has expanded significantly, and various immune system-mediating signaling pathways have been identified as promising targets for enhancing antitumor responses in patients facing aggressive malignancies [2].

Cytokine-induced killer (CIK) cells are characterized by the co-expression of the T-cell marker CD3 and the natural killer (NK) cell marker CD56. These cells can be expanded from human peripheral blood mononuclear cells (PBMCs) through *in vitro* stimulation with interferon-gamma (IFN-γ), anti-CD3 antibody, and interleukin-2 (IL-2) [3]. CIK cells have been investigated as a therapeutic approach for various malignancies, ranging from hematological cancers to solid tumors, including hepatocellular carcinoma (HCC). Moreover, CIK cells have demonstrated low toxicity and a reduced incidence of graft-versus-host disease (GVHD) in allogeneic transplantation settings [4,5].

Clinical studies have demonstrated that the application of CIK cells in cancer immunotherapy significantly improves overall survival (OS) and progression-free survival (PFS) in patients when administered either as a monotherapy or in combination with other therapeutic modalities [6,7]. Prof. Schmidt-Wolf et al., reported the first successful generation of CIK cells from PBMCs under *in vitro* conditions. They subsequently evaluated the antitumor efficacy of these cells in a severe combined immunodeficient (SCID) mouse lymphoma model, followed by their application in human cancer therapy. Studies have demonstrated that CIK cells predominantly mediate cytotoxic effects through interactions between NKG2D receptors and their ligands on tumor cells, leading to the release of pro-inflammatory cytokines such as IFN-*γ* [[8], [9], [10]].

Viruses have been investigated as potential anticancer agents for more than a century [11]. Oncolytic virotherapy, a form of cancer immunotherapy, employs naturally occurring or genetically modified viruses to selectively infect and eliminate tumor cells while sparing normal tissues. Observations of tumor regression during viral infections date back to the 18th century, when researchers such as Václav Trnka and Henri François Le Dran reported cancer remission following severe infectious diseases accompanied by high fever and inflammation [12]. Similar observations persisted into the 19th and 20th centuries, including a case of transient leukemia remission in a 42-years-old woman in 1986 following viral infection [13], as well as multiple reports of tumor regression associated with natural viral infections [11].

Despite these early observations, limited medical knowledge and technical constraints hindered their translation into viable therapeutic strategies, leading to a stagnation in the development of oncolytic viruses (OVs). Subsequent advances in genetic engineering revitalized the virotherapy field. In 1991, Martuza et al., demonstrated that a genetically modified herpes simplex virus type 1 (HSV-1) could inhibit glioma progression in murine models, representing a significant breakthrough [14]. Numerous genetically engineered OVs are currently undergoing clinical evaluation, with promising outcomes, thereby establishing oncolytic virotherapy as a significant and emerging modality in cancer immunotherapy [15,16].

Given the previous literature and clinical findings, Combination therapy strategies are increasingly recognized as critical therapeutic modalities for enhancing the efficacy of cancer treatment [17]. To date, several studies have demonstrated that the combination of OVs and CIK cells can synergistically enhance their antitumor efficacy [18,19]. Although OVs and CIK cells have been individually investigated as promising cancer immunotherapies, the therapeutic potential of their combination has not been comprehensively reviewed. Previous studies have mainly focused on OVs, CIK cells, or other immune-cell-based OV delivery strategies separately, leaving the specific synergistic mechanisms, and translational potential of the CIK cells-OVs combination insufficiently explored. Therefore, this review provides an integrated overview of CIK cells–OVs combination therapy, focusing on its underlying mechanisms, preclinical and clinical evidence, advantages compared with other OV delivery approaches, and current challenges for clinical translation.

## Methodology

In this narrative review, a comprehensive literature search was conducted to identify relevant preclinical and clinical studies published up to June 2026. Literature was retrieved from major scientific databases, including PubMed, Web of Science, and Scopus. The search strategy was developed using a combination of keywords and Medical Subject Headings (MeSH) terms related to cytokine-induced killer (CIK) cells, oncolytic viruses, and cancer. The main search terms included “cytokine-induced killer cells”, “CIK”, “oncolytic virus”, “oncolytic virotherapy”, “OV”, “cancer”, “malignancy”, “neoplasm”, “solid tumor”, “immunotherapy”, and their corresponding MeSH terms. These keywords were combined using Boolean operators (AND/OR) to identify studies investigating the therapeutic potential and combination effects of CIK cells and oncolytic viruses in different cancer models. The identified articles were evaluated based on their relevance to the main topic of this review. Preclinical studies, clinical studies, and relevant review articles focusing on the interaction between CIK cells and OVs in cancer immunotherapy were considered for inclusion. Studies unrelated to cancer immunotherapy, studies not involving CIK cells or oncolytic viruses, and articles lacking sufficient relevance to the scope of this review were excluded.

## Biology and proposed mechanisms of CIK cells and OVs therapies

### CIK cells

CIK cell-based therapy is a form of adoptive cellular immunotherapy that was first described in the 1990s [20]. CIK cells are generated from PBMCs, resulting in a heterogeneous population of immune effector cells. The principal subsets include conventional T lymphocytes (CD3^+^CD56^−^), NK-like T cells (CD3^+^CD56^+^), and natural killer (NK) cells (CD3^−^CD56^+^). Within the CD3^+^T-cell compartment, CD8^+^ cytotoxic T lymphocytes (CTLs) constitute the predominant population, alongside smaller subsets of CD4^+^ helper and regulatory T cells [[21], [22], [23]]. The antitumor activity of CIK cells is primarily mediated by NK-like T cells, which are predominantly CD8^+^, with a minor CD4^+^fraction.

Tumor recognition by CIK cells is through three principal mechanisms. First, their polyclonal T-cell receptors (TCRs) enable major histocompatibility complex (MHC)-restricted recognition of tumor antigens; Second, CIK cells express multiple NK-associated receptors, including NKG2D, DNAM-1, and NKp30, which facilitate MHC-unrestricted recognition of malignant cells [24,25]; among these, NKG2D is considered the most functionally significant [26]. Activation through both NKG2D and TCR signaling is further enhanced by lymphocyte function-associated antigen-1 (LFA-1), which promotes adhesion to tumor cells expressing intercellular adhesion molecules (ICAM-1, −2, and −3) [4,27]. Finally, a subset of CIK cells expresses the Fcγ receptor CD16, enabling antibody-dependent cell-mediated cytotoxicity (ADCC) [28,29].

Upon recognition of tumor cells, CIK cells exert cytotoxic effects through the release of cytolytic granules containing perforin and granzymes or *via* engagement of death-inducing ligands, including Fas ligand (FasL) and tumor necrosis factor–related apoptosis-inducing ligand (TRAIL) [29]. Additionally, these cells secrete pro-inflammatory cytokines, such as tumor necrosis factor-alpha (TNF-*α*) and IFN-*γ*, which further amplify antitumor immune responses [30].

A major advantage of CIK cell therapy is the relative simplicity and cost-effectiveness of its generation. CIK cells are expanded from patient-derived PBMCs, which are first isolated by density-gradient centrifugation and subsequently stimulated with IFN-*γ* to induce a type 1 T-cell phenotype [31]. After 24 h, an anti-CD3 monoclonal antibody and IL-2 are added to promote mitogenic activation, as well as T-cell survival and proliferation [32]. To sustain cell expansion, fresh IL-2–supplemented medium is added every 3–4 days. Although interleukin-1 (IL-1) was included in early CIK culture protocols, it is not commonly used in current clinical-grade production systems [8]. The final CIK cell product is typically ready for infusion after 14–21 days of culture [31]. This standardized protocol yields a cell population comprising >90% CD3^+^T cells, with CD3^+^CD56^+^NK-like T cells accounting for approximately 8–65% [[33], [34], [35]]. Notably, many preclinical and clinical studies have utilized fetal bovine serum (FBS)-supplemented media for CIK cell expansion [[36], [37], [38], [39]].

For clinical use of CIK cells, rigorous quality-control testing is mandatory and typically includes assessment of bacterial, fungal, and mycoplasma contamination, as well as endotoxin levels, to ensure product sterility and safety. In addition, the immunophenotype of the expanded CIK population is routinely evaluated by multiparameter flow cytometry, with particular attention to the frequencies of CD3⁺, CD56⁺, and CD3⁺CD56⁺ cells. Functional potency is frequently confirmed through *in vitro* cytotoxicity assays against tumor target cells, commonly employing colorimetric methods. While many clinical trials implement the full panel of these quality-control tests, others perform only selected assays according to specific protocol requirements or regulatory guidance [[40], [41], [42]].

Although the use of autologous immune cells has shown promising results in cancer therapy, the high heterogeneity of tumor antigens and the profoundly immunosuppressive TME in solid tumors remain major challenges, particularly by limiting effector cell infiltration, persistence, and antitumor activity within malignant and invasive cells in tumor tissues [43].

### OVs

Oncolytic virotherapy represents a rapidly advancing and innovative approach to cancer treatment, in which viruses selectively target and lyse malignant cells while sparing normal tissues [44,45]. Early investigations in the 20th century primarily examined the anticancer effects of wild-type or naturally occurring viruses, including West Nile virus, rabies virus, yellow fever virus, and hepatitis viruses, with their therapeutic potential initially attributed to inherent lytic properties [11]. By the early 2000s, advances in genetic engineering enabled extensive modification of wild-type viruses, facilitating the development of more precise and effective oncolytic platforms [45].

OVs can be classified based on their genomic composition into single- or double-stranded DNA or RNA viruses. Among clinically studied OVs, single-stranded RNA (ssRNA) and double-stranded DNA (dsDNA) viruses are the most prevalent, although *reovirus* (double-stranded RNA, dsRNA) and parvovirus (single-stranded DNA, ssDNA) also represent important categories. dsDNA OVs predominantly include adenoviruses, vaccinia viruses, and herpesviruses. In contrast, ssRNA OVs are subdivided into (i) positive-sense viruses, such as coxsackievirus, Seneca Valley virus, and poliovirus; and (ii) negative-sense viruses, including measles virus, Newcastle disease virus, and vesicular stomatitis virus. Positive-sense ssRNA viruses can be directly translated by host ribosomes, whereas negative-sense ssRNA viruses require prior transcription into positive-sense RNA before translation. Additionally, OVs can be categorized as either naturally attenuated strains or genetically engineered viral vectors based on their structural and functional modifications [46].

OVs exhibit high tumor selectivity by exploiting cancer-specific genetic and microenvironmental abnormalities across multiple cellular compartments. In the extracellular milieu, OVs induce localized cytokine release, disrupt tumor vasculature, and utilize stromal proteases to facilitate viral dissemination. Viral entry is mediated through binding to overexpressed or aberrantly expressed receptors on the surface of cancerous cells, while genetic engineering strategies further enhance targeting specificity. Inside the cytosol, dysregulated antiviral defense mechanisms in cancer cells, particularly impaired protein kinase R (PKR)-interferon (IFN) signaling associated with oncogenic pathways such as RAS, render malignant cells highly permissive to viral replication, whereas normal cells efficiently clear the infection.At the nuclear level, uncontrolled cell-cycle progression and elevated nucleotide pools support the replication of gene-deleted OVs, often further regulated by tumor-specific promoters [47]. In addition to their direct cytolytic activity, oncolytic adenoviruses (OAds) induce immunogenic cell death (ICD), leading to the release of TAAs, pathogen-associated molecular patterns (PAMPs), and damage-associated molecular patterns (DAMPs) [48].

OVs tumor specificity is mediated by distinct viral genes that exert antitumor effects, whereas the viral capsid serves as a nanoscale vehicle for the delivery of nucleic acids [49]. In general, OVs achieve their selectivity by exploiting cell surface receptor expression and intracellular molecular alterations that arise during tumorigenesis and malignant progression [50].

These immunostimulatory signals promote the recruitment and activation of antigen-presenting cells (APCs), enhance T-cell priming, and may convert immunologically “cold” tumors into immune-active phenotypes [51]. This enhanced immune response contributes to tumor regression and supports the establishment of long-term antitumor immune surveillance [52].

OV-induced cell death is associated with the release or surface exposure of potent immunostimulatory molecules, including alarmins such as high-mobility group box 1 (HMGB1), calreticulin, adenosine triphosphate (ATP), and heat shock proteins (Hsp70 and Hsp90), which activate innate immune responses [[53], [54], [55]]. In addition, adenovirus-infected tumor cells secrete a range of cytokines, including TNF-α and type I interferons (IFNs), as well as chemokines such as CXC chemokine ligand 10 (CXCL10). These factors collectively recruit innate immune cells including neutrophils, macrophages, NK cells, and dendritic cells (DCs) to the tumor microenvironment [48]. This cytokine-chemokine milieu establishes a pro-inflammatory environment that promotes tumor elimination, with recruited innate immune cells becoming activated and actively contributing to antitumor responses [48].

### Synergistic mechanisms in CIK cells-OVs combination therapy

The use of CIK cells as cellular carriers for oncolytic vaccinia viruses as the therapeutic payload represents a cell-based combination strategy with the potential to generate additive or synergistic antitumor effects [56]. To date, only a few oncolytic viruses have been successfully delivered to tumor sites using CIK cells as carrier vehicles (Table 1). Consequently, it remains uncertain whether CIK cells can effectively transport additional oncolytic virus platforms beyond those evaluated to date.

**Table 1:** Delivered oncologic viruses with CIK cells as carrier.

OV Platform	Genome (Type/Size)	Replication Kinetics / Site	Immunogenicity & nAb Susceptibility	Safety Profile	Advantages	Key Limitations for CIK Use	Selected References
*Adenovirus*	dsDNA / ∼36 kb	Fast / Nucleus	High / High pre-existing immunity	Good (engineered attenuations)	Easy genetic manipulation; high titers;	High nAb sensitivity; liver tropism	Zhi Yang et al. [73]
*Vaccinia Virus*	dsDNA / ∼190 kb	Rapid / Cytoplasm	High / Moderate	Excellent (smallpox vaccine history)	Large payload for arming (cytokines); rapid spread; strong immune activation	Potential uncontrolled spread	Steve H. Thorne et al. [56]
*Reovirus*	dsRNA / ∼23 kb	Variable / Cytoplasm	Low-Moderate / Moderate	High	Natural Ras-dependent selectivity; systemic potential	Variable clinical efficacy	Xing Zhao et al. [58]
*Measles Virus*	ssRNA(-) / ∼16 kb	Moderate / Cytoplasm	Low-Moderate / High in vaccinated	Good	CD46 targeting (overexpressed on tumors); immune activation	Pre-existing immunity in population	Chunsheng Liu et al. [87]

Accumulating evidence from animal models has demonstrated that CIK cells have the ability to selectively migrate toward tumor tissues. The trafficking and localization of CIK cells within tumors are influenced by multiple factors, including their immunological characteristics, tumor vascularization, and the expression profiles of tumor-associated chemokines and their corresponding receptors [4,5]. These findings were further supported by another *in vivo* study, which showed that CIK cells exhibited effective cytotoxic activity against FasL-expressing malignant cells as well as multidrug-resistant tumor cells. Following systemic infusion, a subset of CIK cells was detected at tumor sites within 72 h and persisted for approximately 9 additional days. During this period, these cells significantly inhibited and eliminated transplanted tumor cells in mouse models [57]. The natural tumor-homing capacity of CIK cells under *in vivo* conditions, together with their intrinsic antitumor activity, supports their potential application as effective cellular carriers for targeted delivery of OVs to tumor sites.

The use of CIK cells as cellular carriers for OV delivery may provide an additional advantage by shielding viruses from neutralizing antibodies present in the circulation. This protective effect can improve the persistence and availability of OVs during systemic administration. For example, Zhao et al. demonstrated that CIK cells could protect oncolytic *reovirus* from antibody-mediated neutralization, allowing the virus to remain viable during circulation in an *in vivo* model [58].

OVs can enhance tumor susceptibility to CIK cell-mediated antitumor activity through several complementary mechanisms. First, OVs induce ICD, which plays a critical role in stimulating antitumor immunity. Following OV infection, tumor cells undergo inflammatory forms of cell death that promote immune activation and enhance tumor recognition [59,60].

Unlike conventional non-immunogenic cell death pathways, ICD is associated with the release of DAMPs, including calreticulin, HMGB1, and ATP, along with the exposure or release of TAAs. These immunostimulatory signals facilitate immune cell recruitment, improve tumor antigen presentation, and promote the activation of antitumor immune responses, thereby potentially enhancing CIK cell-mediated tumor elimination [10].

The OVs have been shown to enhance the expression of tumor recognition ligands on malignant cells. For example, Chen et al. demonstrated that infection with an oncolytic measles virus significantly upregulated the NKG2D ligands MICA and MICB in HCC cells [61]. This increased ligand expression promoted the recognition and cytotoxic activity of CIK cells through the NKG2D receptor, thereby enhancing direct tumor cell targeting.

Another mechanism through which OVs can enhance CIK cell-mediated tumor elimination is through remodeling of the TME. The extracellular matrix (ECM) plays a critical role in regulating tumor progression, invasion, metastasis, and therapeutic resistance [[62], [63], [64]]. However, the dense and immunosuppressive nature of the ECM can limit immune cell infiltration and restrict the penetration and distribution of therapeutic agents within solid tumors.

OVs engineered to express ECM-degrading enzymes, such as collagenase, matrix metalloproteinases (MMPs), and hyaluronidase, have demonstrated considerable potential to overcome these physical and biological barriers [65]. Furthermore, ECM remodeling induced by OVs can contribute to vascular normalization, resulting in enhanced therapeutic agents distribution and improved oxygenation within the tumor core [66]. Consequently, these alterations in the TME promote more efficient infiltration and activity of CIK cells at tumor sites, enabling improved recognition and elimination of malignant cells, including cancer stem cell populations, which represent one of the major challenges in current cancer therapy. Finally, another important mechanism underlying the synergistic effect of OVs and CIK cells in cancer therapy is the induction of cytokine release within the tumor microenvironment.

**Fig. 1:**
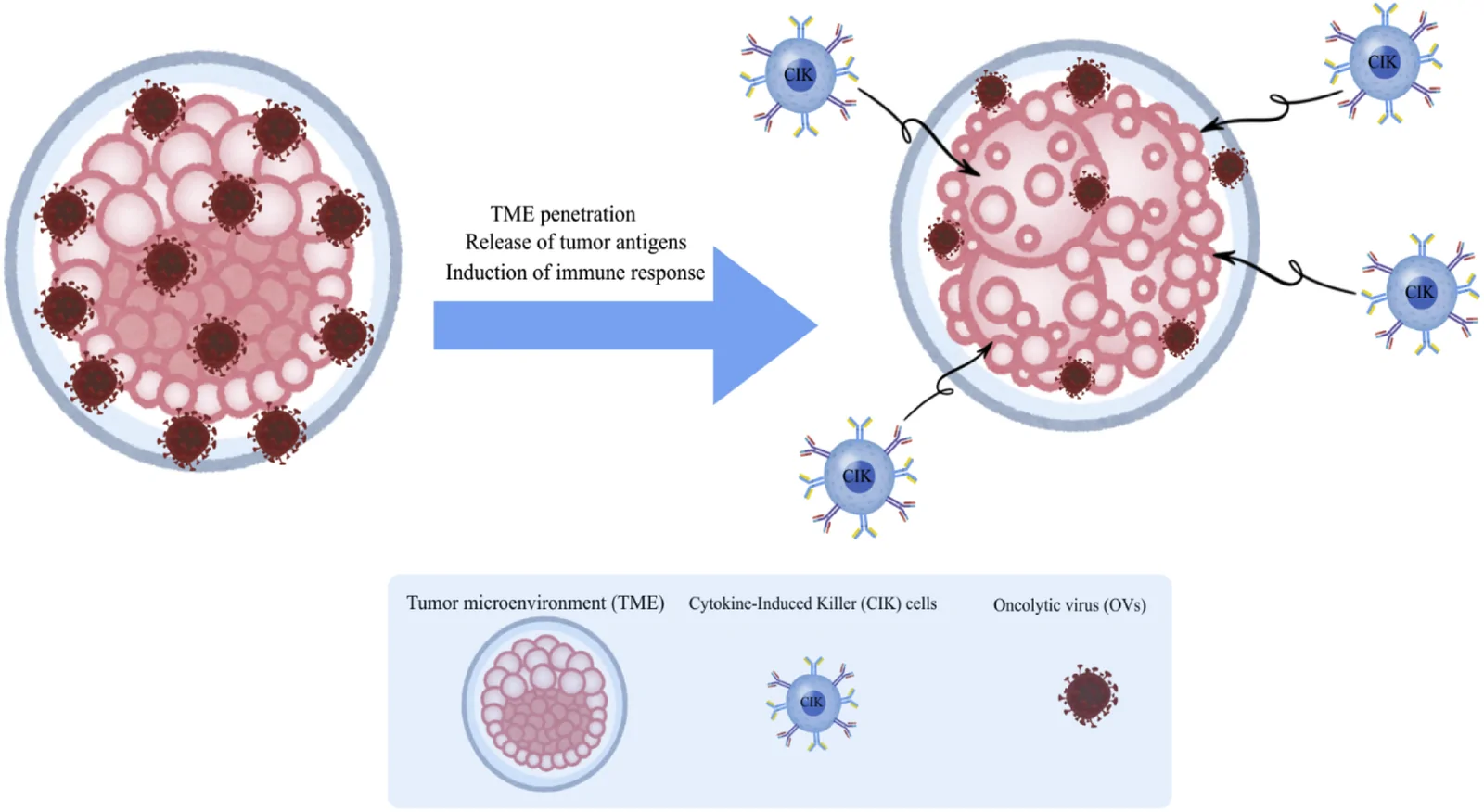
CIK and OV action in the TME.

OV infection can trigger strong innate immune responses and promote the production of pro-inflammatory mediators that enhance antitumor immunity. For instance, infection of murine melanoma cells with oncolytic vesicular stomatitis virus (VSV) rapidly induced the expression of pro-inflammatory cytokines, including IL-6, type I interferons (IFN-I), and TNF-α [67]. Notably, OV-mediated induction of inflammatory cytokines, including IL-6, IFN-I, and TNF-α, may enhance CIK cell-mediated antitumor responses. These cytokines promote immune effector cell activation, proliferation, and cytotoxic function, thereby improving tumor cell elimination [68]. An overview of the underlying mechanisms involved in the combination of CIK cells and OVs is illustrated in Fig. 1.

### Preclinical and clinical evidence

Preclinical studies investigating the combination of OVs and CIK cells can be broadly categorized into two distinct therapeutic strategies: (i) CIK cells serving as carriers for OV delivery (Trojan horse approach), and (ii) CIK cells functioning as an independent immunotherapeutic component administered alongside OVs. These studies are summarized in Table 2.

**Table 2:** Summary of preclinical studies on CIK cells combined with OVs for cancer treatment.

Cancer Type	OV Type	Engineered Payload	CIK Source	Treatment Sequence	Delivery Route	Model System	Main Outcomes	Safety Findings	Major Limitations	Reference
General cancer immunotherapy	*Adenovirus* (CRAd)	IL-12 + IL-15	Not specified (likely human PBMC-derived)	Co-delivery (hydrogel)	Intratumoral (hydrogel co-injection)	*In vitro* (implied) + *In vivo* (suggested, likely xenograft)	Sustained release in tumor; retained therapies; ↓ antiviral response; ↑ antitumor effects with single admin	Attenuated antiviral immune response; reduced off-target dispersion	Hydrogel-specific; translation to humans/clinical scale unknown; limited tumor types tested	Du et al. [18]
Lung cancer	*Adenovirus* ZD55	TRAIL-IETD-MnSOD	Not specified	Combination (CIK + OV)	Not specified (likely systemic/local)	*In vitro* + *In vivo* (likely xenograft)	*In vitro*: ↓ proliferation/invasion, ↑ cytokines (IFN-γ, TNF-α), ↑ LDH. In vivo: ↓ tumor volume/Ki-67, ↑ serum cytokines	Not reported	Preclinical only; specific payload synergy needs broader validation; no long-term safety	Jiang et al. [74]
Liver cancer	*Adenovirus* AdCN205	IL-12	Human PBMC-derived (standard CIK protocol)	Combination (CIK + OV)	Not specified	*In vitro* + *In vivo* (liver tumor model)	↑ cytotoxicity (*in vitro*); significant tumor regression, ↑ survival, ↑ CIK infiltration, ↑ IL-12, ↑ antitumor activity	Not reported in summary	Older study; specific to IL-12 armed virus; potential immunogenicity in clinical translation	Zhi Yang et al. [73]
Various (murine: JC, MC38, 4T1, HCT116)	*Vaccinia* (EEV-enhanced, e.g., WI.TK-)	None (or CCL5 in variants)	Pre-infected CIK cells (murine/human)	Pre-infection of CIK + combination	Systemic? (trafficking enhanced)	*In vivo* (murine models)	↑ tumor trafficking/viral delivery; ↑ antitumor effects, ↓ tumor growth, ↑ survival; CCL5 ↑ homing	Not reported	Murine-focused; virus strain-specific; carrier cell viability post-infection	Sampath et al. [69]
Rectal cancer	*Adenovirus* (E2F-1 regulated, Ad-E2F)	None specified	Not specified	Combination (local)	Local injections	*In vitro* + *In vivo* (orthotopic rectal model)	↑ antitumor effects vs. monotherapies; novel for low rectal cancers	Not reported	Orthotopic model-specific; delivery route invasive; limited generalizability	Xu et al. [70]
TERT-positive tumors	*Adenovirus* (CRAd-CCL21-IL15)	CCL21 + IL-15	Not specified	Combination	Not specified	*In vitro + In vivo*	*In vitro*: apoptosis in TERT+ cells, ↑ CTL cytotoxicity. *In vivo*: ↑ tumor regression vs. monotherapies	Not reported	TERT-specific (not all cancers); payload expression duration?	Ye et al. [72]
Hepatocellular carcinoma	*Adenovirus* AdSurp-Hsp70	Hsp70	CIK-activated (patient-derived?)	Synergistic combination	Not specified	*In vivo* (patient-derived xenograft)	Synergistic tumor inhibition; ↑ CIK chemotaxis/CD3+ infiltration, ↓ Ki67; better in malignant tumors	Not reported	Patient-derived but preclinical; Hsp70-specific; scalability of xenografts	Hu et al. [71]

Abbreviations: ↑, Increase; ↓, Decrease; CIK, cytokine-induced killer cells; TRAIL, Tumor necrosis factor–related apoptosis-inducing ligand; IFN-γ, Interferon-gamma; TNF-α, Tumor necrosis factor-alpha; LDH, Lactate dehydrogenase; IL-12, Interleukin- 12; CCL5, C—C motif chemokine ligand 5;.

In the Trojan horse strategy, Sampath et al. demonstrated that CIK cells preloaded with an EEV-enhanced oncolytic *vaccinia virus* exhibited improved tumor homing and enhanced antitumor efficacy, which was further augmented by viral CCL5 expression. Although this approach provides an innovative solution for systemic OV delivery, the findings were generated primarily in syngeneic murine models, and long-term safety, viral persistence, and definitive evidence distinguishing synergistic from additive therapeutic effects remain insufficiently investigated [69].

Several studies have evaluated CIK cells as a separate immunotherapeutic modality combined with OVs. Yan et al. reported that an E2F-1 promoter-regulated oncolytic *adenovirus* combined with CIK cells significantly inhibited tumor growth in orthotopic colorectal cancer models while exhibiting selective replication in tumor cells and minimal cytotoxicity toward CIK cells. The use of an orthotopic model strengthens the translational relevance; however, limited information regarding administration schedule, comprehensive safety assessment, and validation in immunocompetent models restricts interpretation of the observed synergy [70].

Similarly, Hu et al. demonstrated that a survivin promoter-driven oncolytic *adenovirus* expressing Hsp70 enhanced CIK-cell recruitment and CD3^+^ T-cell infiltration, resulting in superior antitumor activity in patient-derived hepatocellular carcinoma xenografts. Despite the use of clinically relevant patient-derived tumors, the reliance on nude mice with partial immune reconstitution limits evaluation of the complete immune response, while the absence of detailed safety analyses and mechanistic synergy studies remains a notable limitation [71].

Ye et al. developed a TERT promoter-driven conditionally replicating *adenovirus* co-expressing CCL21 and IL-15, which, when combined with CIK cells, produced enhanced apoptosis and tumor regression while sparing normal tissues. Although tumor-selective viral replication and dual cytokine expression represent significant strengths, the immune status of the animal models, treatment schedule, and rigorous analyses distinguishing synergistic from additive cytokine-mediated effects were insufficiently described [72].

Yang et al. combined IL-12-expressing oncolytic *adenovirus* (AdCN205-IL12) with CIK cells in hepatocellular carcinoma models, demonstrating enhanced tumor regression and prolonged survival through improved CIK recruitment. While survival data support the therapeutic potential of this strategy, the use of immunodeficient models and limited evaluation of treatment safety, optimal sequencing, and mechanistic synergy reduce the translational strength of the findings [73].

To address major translational barriers associated with OV dissemination and persistence, Du et al. developed an injectable gelatin-based hydrogel for localized co-delivery of IL-12/IL-15-armed oncolytic *adenovirus* and CIK cells. This platform effectively prolonged intratumoral retention and reduced systemic viral distribution after a single intratumoral administration. Nevertheless, hydrogel-based delivery introduces manufacturing and clinical translation challenges, and validation in more clinically representative models is still required [18].

Finally, Jiang et al. employed CIK cells loaded with engineered ZD55 oncolytic adenoviruses expressing TRAIL and MnSOD for lung cancer therapy. This carrier-based strategy enhanced antitumor efficacy, suppressed tumor proliferation, and increased inflammatory cytokine production compared with either monotherapy. However, conclusions are limited by the use of xenograft models, restricted toxicological evaluation, and the lack of rigorous analyses confirming true synergistic interactions rather than additive therapeutic effects [74].

These studies collectively support the feasibility of OV + CIK combinations, with some evidence of improved trafficking, cytokine amplification, and localized efficacy. However, weaknesses predominate: heavy reliance on xenograft/nude or limited immune-reconstituted models hinders extrapolation to human immunocompetent settings; incomplete reporting on administration timing, routes (often intratumoral), control groups, and statistical rigor for synergy; variable safety data (e.g., minimal CIK cytotoxicity noted, but systemic effects underexplored); and unclear distinction in many cases between CIK-carrier versus effector roles, muddying mechanistic insights. Few studies rigorously test true synergy (e.g., *via* Chou-Talalay or timing-optimized designs). Translational potential is promising for solid tumors (colorectal, HCC, lung, and gastric), but requires better standardization, immunocompetent models, and safety/efficacy benchmarks before advancing combinations.

Although numerous clinical studies have independently evaluated the anticancer efficacy of OVs and CIK cells, to date, only one phase I clinical trial (NCT04282044) has investigated their combined application for the treatment of solid tumors, as outlined below (Table 3).

**Table 3:** Clinical trial of combining OV with CIK cells for advanced solid tumors.

Title	NCT Number	Status	Conditions	Interventions	Phase	Link
Study of CRX100 as Monotherapy and in Combination With Pembrolizumab in Patients With Advanced Solid Malignancies	NCT04282044	Recruiting	Advanced solid tumors including non-small cell lung cancer (NSCLC), ovarian cancer, colorectal cancer, hepatocellular carcinoma (HCC), malignant melanoma (excluding uveal melanoma), gastric cancer, triple negative breast cancer (TNBC), and osteosarcoma	CRX100 (CIK cells combined with oncolytic virus), preconditioning with fludarabine and cyclophosphamide; combination cohorts include pembrolizumab	Phase 1	https://clinicaltrials.gov/study/NCT04282044

This open-label, dose-escalation study evaluates CRX100 (autologous CIK cells loaded *ex vivo* with a tumor-specific oncolytic *vaccinia virus*), administered as monotherapy or in combination with pembrolizumab following preconditioning with fludarabine and cyclophosphamide. The trial targets patients with advanced solid tumors, including non-small cell lung cancer (NSCLC), ovarian cancer, colorectal cancer (CRC), HCC, melanoma, gastric cancer, triple-negative breast cancer (TNBC), and osteosarcoma. As of recent updates, the trial is ongoing, with preliminary data indicating acceptable safety (no dose-limiting toxicities reported in initial cohorts, with mild fever as a common adverse event) and early signs of clinical activity, such as stable disease and biomarker responses in a small subset of heavily pretreated patients (e.g., recurrent platinum-resistant ovarian cancer).

### Challenges and limitations

Clinical evidence supporting the combined use of OVs and CIK cells remains scarce, underscoring the early developmental stage of this therapeutic strategy. Future clinical translation will require carefully designed, standardized trials to establish its safety, tolerability, and therapeutic efficacy. The integration of longitudinal biomarker profiling, immune monitoring, and comprehensive clinical outcome assessments into these studies will be essential for elucidating the mechanistic basis of therapeutic synergy, identifying predictive biomarkers of response, and optimizing patient selection.

While OV-based cancer therapies have demonstrated promising clinical outcomes, including the FDA-approved talimogene laherparepvec (T-VEC) for the treatment of metastatic melanoma, several substantial challenges remain. Key limitations include the presence of a dense extracellular matrix, which acts as a physical barrier restricting viral dissemination; an immunosuppressive tumor microenvironment that impairs antitumor immune responses; difficulties in accurately evaluating therapeutic efficacy (e.g., false-positive rates exceeding 40% in PET-CT imaging); and suboptimal systemic delivery strategies [75,76]. In addition, pre-existing neutralizing antibodies can significantly reduce the therapeutic efficiency of oncolytic viruses. Patients with higher baseline neutralizing antibody titers exhibit a markedly reduced median OS of 12.5 months compared with 21.2 months in patients with lower titers (HR = 2.0) [77]. Beyond neutralization of free virus, host antiviral immunity poses a second, distinct risk specific to the cell-carrier strategy: when CIK cells are used as OV carriers and infected CIK cells begin expressing viral antigens on their surface, they themselves may become targets of innate and adaptive antiviral responses (NK cell and CD8^+^ T cell–mediated clearance).

Oncolytic virotherapy may also induce cytokine release syndrome (CRS), characterized by rapid and substantial elevations in pro-inflammatory cytokines such as IL-6 and TNF-α [78]. CRS is among the most severe acute toxicities associated with OV therapy [79,80]. Despite preventive strategies, including the administration of corticosteroids such as dexamethasone [81] and the use of localized delivery approaches [82], clinical manifestations of CRS, such as pyrexia, elevated aspartate aminotransferase (AST) levels, thrombocytopenia, and treatment interruptions, remain frequently observed [83,84]. Furthermore, off-target effects remain a major concern, particularly for adenoviral vectors, which tend to accumulate predominantly in hepatic tissue, raising the risk of hepatotoxicity [35]. In addition, oncolytic viral therapy induces immunogenic cell death, leading to the release of DAMPs and activation of the cGAS–STING signaling pathway. This process can convert immunologically “cold” tumors (with a PD-1 inhibitor objective response rate of only 38%) into immunologically “hot” tumors, while simultaneously increasing CD103^+^ dendritic cell infiltration from 5% to 25% [85].

In terms of CIK cell–based cancer therapy, several challenges remain. For example, the generation of CIK cells requires good manufacturing practice (GMP)-grade production, which typically takes approximately six to eight weeks depending on quality control and release criteria. This extended manufacturing period raises concerns regarding its alignment with the optimal therapeutic window, particularly in patients at risk of early molecular relapse [86]. In addition, the use of CIK cells as carriers for natural or genetically modified OVs may introduce potential safety concerns, including unintended genetic or functional alterations in CIK cells. Such changes could theoretically affect their biological behavior, potentially leading to malignant transformation or exaggerated immune activation, including CRS in patients. Quality control of the final cell–virus product therefore becomes a distinct translational hurdle beyond that of either component alone: release criteria must verify not only CIK viability, purity, and cytotoxic potency, but also viral titer per cell, absence of replication-competent contaminants (where a replication-defective platform is intended), and batch-to-batch consistency of the infected-cell product, none of which are captured by standard CIK-alone or virus-alone release assays.

In several studies, CIK cells have been employed as cellular carriers for OVs to enhance targeted viral delivery to tumor sites. However, OV loading can influence the biological properties and functionality of CIK cells. For example, Liu et al. demonstrated that MV infection reduced the viability of CIK cells [87]. Furthermore, viral replication within CIK cells may alter their cytokine secretion profile, including the production of TNF-α, IFN-γ, and other immunomodulatory mediators, potentially affecting their antitumor activity. In addition, OV infection may induce transcriptional and phenotypic changes in carrier CIK cells. Although no evidence currently suggests that OV loading results in malignant transformation of CIK cells, the long-term effects of viral replication on CIK cell genomic stability, phenotype, and biological function remain insufficiently characterized and warrant further investigation before widespread clinical application.

Finally, intratumoral heterogeneity, defined as the genetic and phenotypic diversity within individual tumors, further complicates the clinical application of immunotherapeutic strategies [88]. Significant challenges persist in the design, execution, and integration of immunotherapy-based clinical trials [89].

## Conclusion

The combination of CIK cells and oncolytic viruses offers a mechanistically rational strategy for overcoming the trafficking and immunosuppressive barriers that limit cell-based immunotherapy in solid tumors, using CIK cells as both effector and viral delivery vehicles while OVs provide direct oncolysis and immune activation. However, current support for this combination rests almost entirely on preclinical and early mechanistic studies; controlled clinical evidence specific to CIK-OV combinations remains scarce, and efficacy and safety in patients should be regarded as unproven rather than established.

Three barriers will determine whether this approach translates: (i) safety, particularly the risk of CRS and other immune-related adverse events associated with OV administration in combination with adoptively transferred effector cells; (ii) biological heterogeneity, both in CIK cell phenotype/function across preparations and in intratumoral heterogeneity that may drive immune escape; and (iii) the immunosuppressive TME, which can blunt both viral spread and CIK persistence even when delivery barriers are overcome.

Future progress will depend on well-designed, adequately controlled early-phase clinical trials that report safety and immunologic endpoints alongside efficacy, standardized and reproducible protocols for CIK-OV manufacturing, and combination strategies specifically designed to counteract TME-mediated resistance. Until such data accumulate, the CIK-OV approach should be considered a promising but early-stage strategy rather than a near-term clinical option.

## Financial and competing interest

The authors declare that they have no known competing financial interests or personal relationships that could have appeared to influence the work reported in present study.

## Data availability

No datasets were generated or analysed during the present study.

## CRediT authorship contribution statement

**Milad Parvinzad Leilan:** Writing – original draft, Visualization, Investigation, Conceptualization. **Mona Arianejad:** Writing – original draft. **Behnam Omidi Sarajar:** Writing – review & editing. **Gholamreza Tavoosidana:** Writing – review & editing, Supervision, Project administration.

## Declaration of competing interest

The authors declare that they have no known competing financial interests or personal relationships that could have appeared to influence the work reported in this paper.
